# Firecracker eye injuries during Deepavali festival: A case series

**DOI:** 10.4103/0301-4738.60095

**Published:** 2010

**Authors:** Ravi Kumar, Manohar Puttanna, K S Sriprakash, B L Sujatha Rathod, Venkatesh C Prabhakaran

**Affiliations:** Department of Ophthalmology, Minto Ophthalmic Hospital, Bangalore, India

**Keywords:** Deepavali, firework, firecracker injuries

## Abstract

We report a large series of ocular injuries caused by fire-crackers. This study was a hospital-based, singlecenter, retrospective case series in which the records of 51 patients with ocular injuries were analyzed. Injuries were classified according to Birmingham eye trauma terminology system (BETTS). Visual outcomes before and after the intervention were recorded. Ten patients were admitted for further management. As ocular firecracker injuries result in significant morbidity, public education regarding proper use of firecrackers may help in reducing the incidence of ocular injuries.

Firecracker injuries can cause serious and irreparable damage to vision. In India, firecracker injuries are common during the festival of ‘Deepavali’ where traditionally, firecrackers form an essential part of the celebrations.

While a number of papers have dealt with firecracker injuries, very few large case series on this subject exist in literature.[1–3] We report a series of firecracker injuries seen during a single week to highlight the importance of firecrackers as a cause of ocular injuries in India.

## Materials and Methods

This was a retrospective case series. All patients with firecracker injuries who attended the emergency eye care services of a tertiary eye care hospital in South India, during the ‘Deepavali’ festival week (from 26 October to 2 November 2008) were included in this study.

The patients underwent a detailed ocular examination. Ultrasonography (USG) A and B scans, Gonioscopy and fundus photography and X-ray orbit was done as and when indicated. The injuries were classified according to Birmingham eye trauma terminology system (BETTS) [Table 1].

**Table 1:** Glossary of terms used in classification of injuries

Open globe injury	Full-thickness injury of the eye wall
Closed globe injury	No full-thickness injury of the eye wall
Contusion	A closed globe injury due to direct energy delivery to the eye wall, e.g. - Angle recession
Lamellar laceration	Partial thickness injury of eye wall
Laceration	Full-thickness injury of eye wall caused by a sharp object
Penetrating injury	An open globe injury with an entrance wound
Perforating injury	An open globe injury with an entrance and exit wound

Adapted from Kuhn F, Morris R, Witherspoon CD, Heimann K, Jeffers JB, Treister G. A standardized classification of ocular trauma. Ophthalmology 1996;103:240-243

Although patients with closed eye injuries were treated on an outpatient basis, most cases with open eye injury were advised admission for further management and observation. Admitted cases included patients with corneal and scleral tears, traumatic iridodialysis with hyphema, suspected intraocular foreign body (IOFB), and globe rupture.

## Results

**Figure 1:**
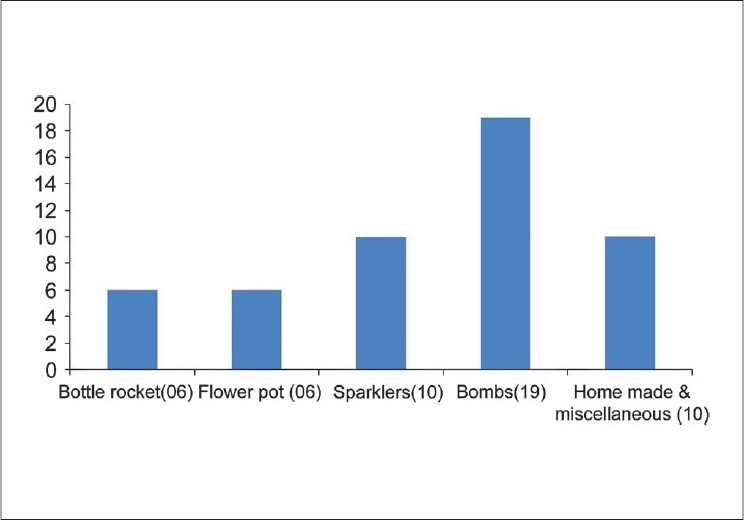
Type of fireworks causing injury

Of the 51 patients seen, 40 were males. The age range of these patients was 3 to 70 years (mean of 19 years). Thirty-one patients were less than 20 years of age. The most common cause of ocular injuries were bombs (37%), followed by sparklers (19%) [Fig. 1]. Bottle rockets and bombs were responsible for the most serious ocular injuries observed in our patients.

**Figure 2:**
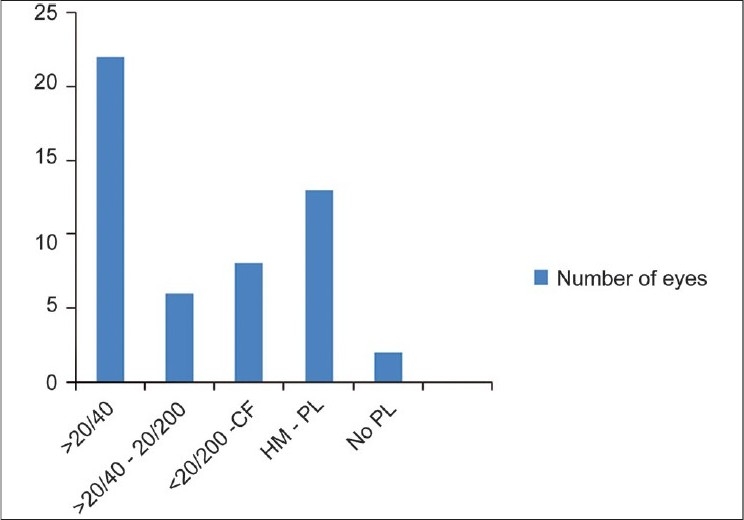
Initial visual acuity of the patients attending ocular emergency department; CF - counting finger, HM - Hand movement, PL - Perception of light

**Figure 3:**
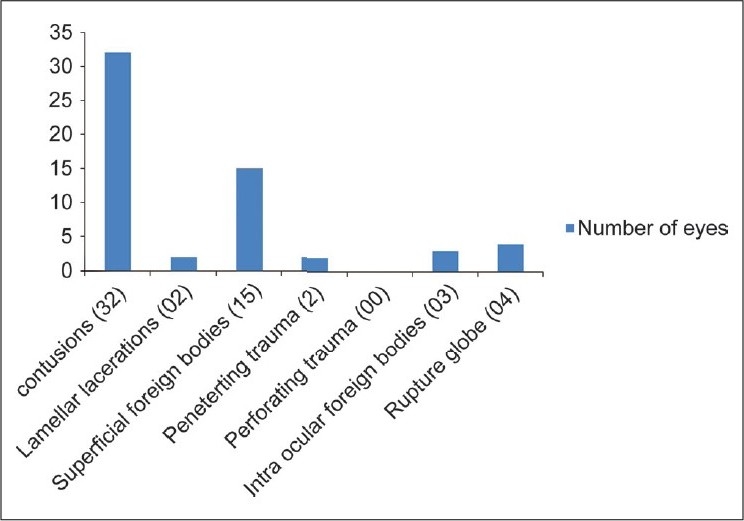
Distribution of eye trauma according to birmingham eye trauma terminology system

**Figure 4:**
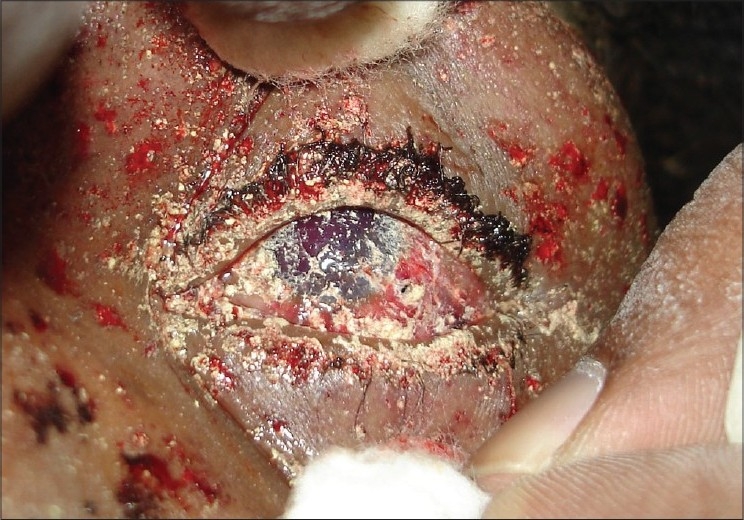
Clinical photograph of a patient with firecracker injuries showing multiple superficial foreign bodies. This patient subsequently underwent amniotic membrane graft for nonhealing corneal ulcers

**Figure 5:**
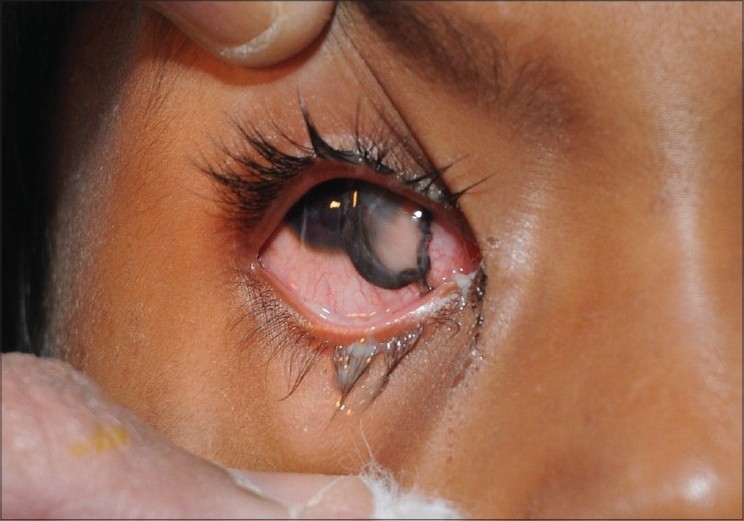
Clinical photograph of a patient with an open globe penetrating firecracker injury. Patient underwent primary scleral tear repair

**Figure 6:**
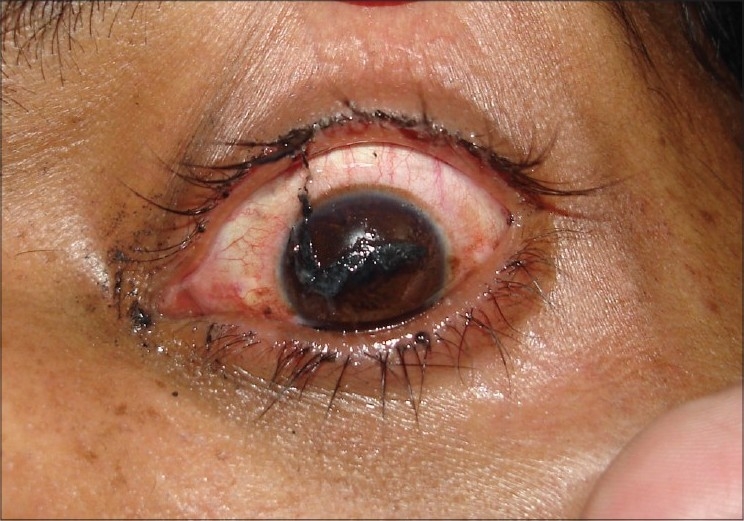
Clinical photograph of a patient with firecracker injuries showing soot particles over cornea following firecracker injury. Fluorescent staining showed extensive epithelial defect. Following treatment patient's visual acuity improved from Snellen 20/200 to 20/40

**Figure 7:**
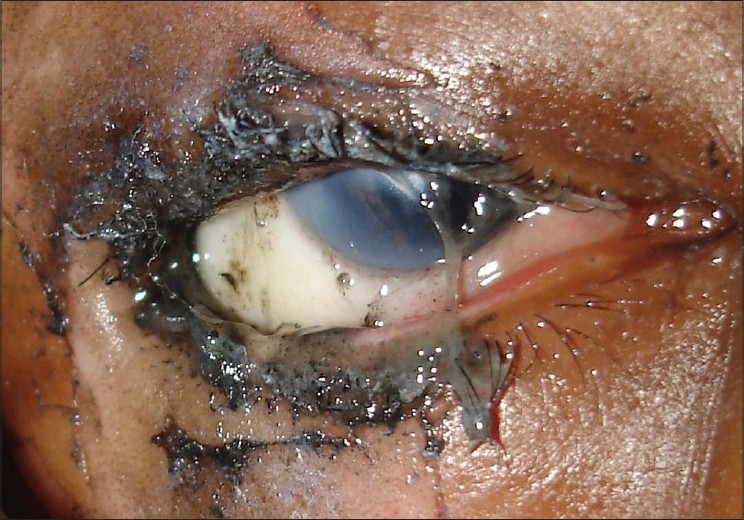
Clinical photograph of a patient with burn injuries due to firecrackers showing superficial lid burns, seething of eyelashes, limbal ischemia in the inferotemporal quadrant and melting of cornea. The cornea shows combined chemical and burn injury

Twenty-nine patients were onlookers and 22 patients were actively involved in igniting the firecrackers. None of the firecracker victims reported using any protective eye wear at the time of injury. The right eye was involved in 31 cases and left eye in 27 cases. According to the initial assessment of vision at the time of presentation to the hospital two eyes of two patients had no perception of light (PL negative), 13 eyes of 11 patients had visual acuity of hand movement to perception of light (PL positive) while eight patients had counting fingers to 20/200 vision [Fig. 2]. Vision of five patients was not recorded (all were less than seven years of age and not cooperative for vision assessment at initial presentation). The distribution of severe eye injury (hand movement-PL negative) was nearly equal in bystanders and actively involved individuals. According to BETTS, nine cases were open globe injuries and 49 cases were closed globe injuries [Figs. 3–7].

In all, 10 patients were admitted to our hospital with firecracker injuries. All patients except one had visual acuity of hand movement or worse at the time of initial evaluation. Three of these patients underwent surgical repair for scleral tear. Two patients underwent surgery for corneoscleral tear repair. One patient required amniotic membrane graft and autologous serum drops for non-healing epithelial defects [Fig. 4]. Visual outcome in all these cases was poor (three patients had no PL and two patients had only PL). One patient with total hyphema had visual recovery following anterior chamber wash from visual acuity of hand movements at presentation to 20/40 Snellen at discharge. Four others were treated conservatively as per the standard treatment protocol for ocular burns and chemical injuries.[4] Injured eyes were irrigated with copious amount of normal saline and particulate matter and soot particles were removed with forceps under local anesthesia; pH was monitored before and after ocular irrigation. Patients were prescribed antibiotic steroid eye drops (tapered after a week), lubricating solution and ointment and cycloplegic eye drops. Later, two patients underwent lid reconstruction for correction of cicatricial entropion.

The average number of days of stay of admitted patients was seven days (median = six days). Though three patients ended up with no PL, most had a moderate visual recovery [Table 2].

**Table 2:** Visual outcome of 12 eyes of 10 admitted patients

Vision	At admission (number of eyes)	At discharge (number of eyes)
>20/40	0	2
20/40 - 20/200	1	2
<20/200 - CF	1	2
HM+	5	1
PL+	4	2
PL−	1	3

CF - Counting finger, HM - Hand movement, PL - Perception of light

## Discussion

This study was a hospital-based, single-center, retrospective case series of firecracker injuries.[3,5–8] The injuries reported ranged from conjunctival or corneal burns to globe rupture with interventions ranging from ocular wash to repair of globe perforation. Most of the patients were below the age of 20 years. Unlike the findings in some studies where victims were mostly those who were actively involved in igniting the firecracker, more than half of the victims in our study were bystanders.[7,9,10] The most common firecracker causing injury in our study were bombs followed by sparklers and homemade devices. Even though sparklers were reported to cause minimal injuries in one of the studies, were not found to be innocent in our study.[5] Most bottle rocket injuries were of a serious nature.

Many of the injuries were caused as a result of negligence of those igniting the firecrackers. Some of the severely injured patients reported device malfunction as the cause of their injury. In three cases, the attempt to reignite or recover a failed device was the cause of injury. In one instance, the patient suffered severe facial and bilateral ocular injuries when he attempted to ignite a homemade device made up of unburnt firecracker powder [Fig. 4].

Ocular injuries by firecrackers are common during ‘Deepavali’. Lack of knowledge about safety measures or not following them was a reason for eventualities. Absence of parental supervision, and failure to maintain safe distance from firecrackers were contributory in some cases of injuries. The other major cause of injury is the common practice of igniting firecrackers in the streets thus exposing passersby to injury.

The fact that so many cases were seen in a single center highlights the enormous health importance of regulating firecracker use and enforcing safety precautions. The single most effective measure may be to restrict the fireworks to public open spaces (such as parks or playgrounds). Regulating the quality of firecrackers and promoting safe use via schools and media will also have a positive impact.
